# Effect of the extraction solvent and method on the determination of the total polyphenol content in different common buckwheat (*Fagopyrum esculentum* Moench) varieties

**DOI:** 10.29219/fnr.v69.9834

**Published:** 2025-01-09

**Authors:** Pavlína Podloucká, Ivana Polišenská, Ondřej Jirsa

**Affiliations:** Agrotest Fyto, Ltd., Kroměříž, Czech Republic

**Keywords:** total polyphenol content, buckwheat, solvent extraction, acid hydrolysis, alkaline hydrolysis

## Abstract

Buckwheat is a pseudocereal whose seeds are rich in numerous health-positive phytochemicals including polyphenols. Several methods for extracting these compounds can be found in the literature. The objective of the study was to compare the total polyphenol content (TPC) of seven common buckwheat (*Fagopyrum esculentum* Moench) varieties obtained using different extraction methods/procedures and to assess the impact of the extraction methods on various varieties. The Folin–Ciocalteu spectrophotometric assay was used to measure the TPC. The results showed that TPC was significantly dependent on the extraction solvent and the efficiency of the solvents may be ordered from high to low efficacy as follows: 80% acetone > 0.1% HCl in methanol > 80% methanol = 80% ethanol > 100% methanol > water = 100% ethanol. TPC increased with increasing temperature during extraction and procedures based on alkaline hydrolysis proved to be more effective than those based on the acidic one. The responses of different buckwheat varieties to various extract preparations slightly differed, which could be attributed to each variety’s specific composition of extractable and bound polyphenols. It can be suggested that using more than one extraction method gives more robust information for good characterizing of buckwheat varieties according to their TPC for breeding and food use purposes.

## Popular scientific summary

- Buckwheat is a crop which is rich in health-positive phytochemicals including polyphenols. To measure the total polyphenol content (TPC) of seven varieties of common buckwheat, the Folin-Ciocalteu spectrophotometric method was used. The results showed that TPC significantly depended on the extraction solvent and the extraction procedure. The response of different varieties to various extract preparations slightly differed, which could be attributed to each variety’s specific composition of extractable and bound polyphenols.

Current research and epidemiological studies show that a diet rich in fruits, vegetables, and different seeds has a positive effect on human health and can reduce the risk of developing chronic diseases common in modern civilization. Fruits, vegetables, and seeds are rich sources of dietary fibre and antioxidant compounds, which are mainly responsible for their positive effects on humans. Research is often focused on a large group of compounds known as polyphenols, which have an ideal antioxidant structure due to the presence of an aromatic phenolic ring that stabilizes an unpaired electron (1, 2). In recent decades, polyphenols have attracted interest because of the link between their intake and the prevention of certain chronic diseases (2–4). Polyphenolic compounds are secondary metabolites of plants that can act as mechanical support for plant growth. They play an important role in pollinator attraction or plant protection from UV radiation, pathogens, and pests. In addition, they affect the colour, taste, and digestibility of plant nutrients. Generally, the polyphenol group contains several thousands of molecules with a wide range of chemical structures from small molecules, such as phenolic acids, to large complex polymers, such as proanthocyanins. Polyphenols may also be linked to carbohydrates, proteins, and lipids (5).

The assays used for the analysis of phenols can be classified as either those that determine total phenolic content or those quantifying individual phenols or phenol-specific groups. However, compounds must first be extracted from the studied matrix. Generally, in the extraction method, solvents are chosen according to extraction purposes (i.e. the desired compounds to extract and reasons to extract them), the physicochemical properties of the matrix (i.e. the solubility and polarity of the compound in a given solvent), the availability of chemicals and equipment, cost, and safety (6). The structural diversity of polyphenols leads to variability in their physicochemical properties, which may affect their extraction and their subsequent study. Przybylski et al. proved that the amount of extracted phenolic compounds increases with the increasing polarity of the extracting solvent (7). A range of solvent extraction methods can be found in literature (8). Most frequently, aqueous mixtures of organic solvents (ethanol, methanol, or acetone) are used. These solvents preferentially extract phenolic compounds with a low molecular weight which are free or conjugated but soluble in an aqueous-organic solvent, that is, phenolic acids and flavonoids. Hydrolysis must be used to release polyphenols with a high molecular weight or bound phenolic compounds in cell components (e.g. proanthocyanins, bounded phenolic acids, and hydrolyzable tannins). The number of possible solvents and the diversity of the methods complicates the comparison of results from different studies.

Buckwheat belongs to the family *Polygonaceae*. It is classified as a pseudocereal because its seeds have similar uses and chemical composition as cereals. For many years, the cultivation of buckwheat has been in decline, but lately, interest in this crop has increased because of its health-promoting properties. In addition, buckwheat has various advantages from the cultivation viewpoint, such as a short growing season and ease of growth. Seeds have high nutritional value, and they are a source of numerous health-positive phytochemicals, including polyphenols. The primary polyphenols in buckwheat are rutin, quercetin, hyperin, and catechins (9). Whole buckwheat contains more phenolic compounds than cereals (10, 11). Some health benefits attributed to buckwheat polyphenols include prevention against cardiovascular and ageing diseases, as well as neuroprotection, anticancer, anti-inflammatory, and antidiabetic effects (3, 12, 13).

The aim of the study was to clarify how different solvents and different extraction methods would affect the yields of total polyphenol content (TPC) in buckwheat. And further, whether the potential differences would be the same for various common buckwheat (*Fagopyrum esculentum* Moench) varieties. The simplest possible extraction procedures with no need for any special equipment together with seven different solvents (100 and 80% methanol, acid methanol, 100 and 80% ethanol, water, and 80% acetone) have been used for TPC analysis in seven common buckwheat varieties. Also, the effects of temperature and two hydrolysis procedures (classical and sequential) were tested.

## Materials and methods

### Chemicals

All chemicals were of analytical reagent grade; methanol and water were high-performance liquid chromatography grade. The Folin–Ciocalteu reagent and Na_2_CO_3_ were obtained from Penta Chemicals (Prague, Czech Republic). Methanol and water were obtained from Chem-Lab NV (Zedelgem, Belgium), and HCl and NaOH were from MicroCHEM (Pezinok, Slovak Republic). Gallic acid was obtained from Sigma-Aldrich Chemie GmbH (Taufkirchen, Germany).

### Plant material

For this study, seven varieties of common buckwheat (Darja, Harpe, Kora, Panda, Zamira, Zita, and Zoe) were used (Table 1). The six varieties were grown and harvested in 2017 at experimental fields at OSEVA Development and Research in Zubří, Czech Republic, and the seventh variety (Darja) was obtained from Slovenia (the 2017 harvest). Postharvest, the grains were dried and stored in a cool, dry place. The whole, unhulled buckwheat seeds were ground just before extraction using a laboratory mill VM7 (OPS Přerov, Czech Republic).

**Table 1:** Information about used varieties of common buckwheat (*Fagopyrum esculentum* Moench)

Variety	Year of registration	State of registration	Thousand grain weight (g)	Growing period (days)	Earliness	Interesting properties
Darja	1988	Slovenia	Medium		Late	Fast-growing, robust and versatile
Harpe	Before 1990	France	19–22	100–120	mid-late	Good resistance to diseases, and it is mainly grown to make flour
Kora	1989	Poland	25–30	85–95	Early/ mid-early	Good resistance to spring frosts, drought, and lying down and diseases
Panda	1998	Poland	25–32	90–105	Early/ mid-early	Good resistance to diseases, drought, and spring frost
Zamira	2014	Czech Republic	~ 30	110–115	Early/ mid-early	Good resistance to disease
Zita	2009	Czech Republic	~ 33	110–120	Early/ mid-early	Good resistance to biotic and abiotic stress
Zoe	2010	Czech Republic	~ 29	100–115	Early	Good resistance to diseases

### Extract preparation

1) The procedure used for polyphenol extraction was published by Eliášová et al. (14), and it was performed both with and without modifications. In test tubes, 0.5 g of ground seeds were extracted with 5 mL of 0.1% HCl in methanol. One tube was incubated overnight at −20°C (original procedure), and the second was left at ambient temperature in the dark (modified procedure). During this time, the mixtures were vortexed several times. The next day, the samples were centrifuged (10 min, rcf = 10,487) and supernatants were analyzed. To compare solvent influence, 100 and 80% methanol, 100 and 80% ethanol, water, and 80% acetone were also used in the modified procedure.

2) The hydrolysis extraction procedure was slightly modified from the procedure published by Stratil et al. (15). Briefly, 0.5 g of ground seeds were added into three tubes. About 10 mL of 80% methanol, 10 mL of acidified methanol (1:1; 2.4 mol/L HCl and methanol), or 10 mL of alkaline methanol (1:1; 2 mol/L NaOH and methanol) was added to tubes. Tubes were incubated at 80°C for 120 min and vortexed every 30 min. Subsequently, the mixtures were cooled to ambient temperature. The mixture with methanol was refilled to 10 mL with 80% methanol. The mixture with acidified methanol was refilled up to 20 mL with 80% methanol. Finally, the mixture with alkaline methanol was neutralized by acidified methanol and then refilled up to 20 mL. This was followed by centrifugation for 10 min, and then, supernatants were analyzed.

3) Sequential hydrolysis was divided into two steps. First, 5 mL of 80% methanol was added to two tubes. They were vortexed and allowed to stand in the dark at room temperature overnight. The next day, they were centrifuged for 10 min (rcf = 10,487), and the first supernatants were separated. Ten mL of acidified methanol (1:1; 2.4 mol/L HCl and methanol) or 10 mL of alkaline methanol (1:1; 2 mol/L NaOH and methanol) was poured into the rest of the tubes. The procedure was repeated as described earlier in the text. After cooling to ambient temperature, the alkaline mixture was neutralized and the first supernatant was added to the solution. The mixture was pooled into 25 mL. The first supernatant was added to the mixture with acidified methanol, and the mixture was pooled into 20 mL.

### Determination of TPC

The TPC was determined by a modified Folin–Ciocalteu spectrophotometric assay, which is based on the reduction of the phosphowolframate–phosphomolybdate complex by phenols (16, 17). This method is widely used for the analysis of TPC. Its advantages include simplicity, speed and high reproducibility. However, the reagent is not specific only for phenolic compounds and it can react with other oxidizable compounds, that is, vitamin C, aromatic amines, reducing sugars, and aromatic amino acids.

To 0.125 mL of the sample extract, 0.375 mL of redistilled water was added into a test tube. Then, 0.250 mL of Folin–Ciocalteu reagent, 8 mL of distilled water, and 1.250 mL of 20% (w/w) Na_2_CO_3_ were added. The total volume was 10 mL. The test tube was shaken and allowed to stand in the dark at room temperature for 2 h. Absorbance was measured twice for each sample using a Jena spectrophotometer Spekol 11 (Carl Zeiss, Oberkochen, Germany) at 760 nm against a blank (redistilled water). Gallic acid was used as a standard for calibration curve construction, and the results were expressed as the gallic acid equivalent (GAE) per 1 g of the buckwheat sample. Four replications were carried out for each sample.

### Data analyses

The TPC is expressed as the mean value of four replications ± standard deviation. The significance of the differences in TPC obtained for different solvents and procedures and between buckwheat varieties was tested using analysis of variance (ANOVA). Statistical comparison of the means was made using the post-ANOVA Tukey’s honestly significant difference test when significant main effects were detected. The significance level was set at *P* < 0.05. For statistical analysis of differences between buckwheat varieties, the data of the Darja variety were omitted because it was grown at a different location, and the possible influence of environmental conditions could not be excluded. All calculations were performed using the software package Statistica version 12 (StatSoft Inc., Tulsa, OK, USA).

## Results and discussion

### Effect of the solvent

To study the influence of the solvent on the amount of extracted total polyphenols, our research tested seven different solvents, that is, 100 and 80% methanol, acid methanol (0.1% HCl in methanol), 100 and 80% ethanol, water, and 80% acetone. The extraction process is described in the section ‘Materials and methods’ (Extract preparation, paragraph 1). The phenolic compounds found in such extracts are the so-called ‘extractable phenols’ and are free or soluble conjugated polyphenols. It should be noted here that each solvent may be employed to extract only free and soluble conjugate polyphenols whereas bound phenols may eventually be released only by alkaline, acid, or enzymatic hydrolysis. This is discussed later.

The results of the TPC obtained by different solvent extractions are summarized in Table 2. The results clearly showed that polyphenol extraction was significantly affected by the solvent used. For all varieties of buckwheat, the most effective solvent was 80% acetone, and the least effective was 100% ethanol. When acetone was used, the TPC in extracts was nearly three times higher (and in the case of variety Zoe, eight times higher) than that in extracts obtained by 100% ethanol. The TPC in buckwheat seeds extracted in 80% acetone ranged from 5.51 (Kora) to 8.43 mg GAE/g (Darja). TPC values in 100% ethanol ranged from 0.85 (Zoe) to 2.79 mg GAE/g (Harpe).

**Table 2:** The total polyphenol content of seven varieties of common buckwheat in different solvents (mg GAE/g). Results are expressed as means of four replications ± SD

	TPC (mg GAE/g)						
	Darja	Harpe	Kora	Panda	Zamira	Zita	Zoe
80% methanol	4.80^c^ ± 0.04	4.12^b^ ± 0.12	3.44^b^ ± 0.10	4.13^b^ ± 0.23	3.79^d^ ± 0.09	4.11^d^ ± 0.26	3.20^d^ ± 0.23
100% methanol	4.11^b^ ± 0.13	3.86^b^ ± 0.05	3.82^b^ ± 0.28	3.96^b^ ± 0.17	3.38^c^ ± 0.17	3.45^c^ ± 0.05	2.48^c^ ± 0.12
0.1% HCl in methanol	5.71^d^ ± 0.12	5.50^c^ ± 0.13	4.59^c^ ± 0.15	5.41^c^ ± 0.20	4.95^e^ ± 0.21	5.38^e^ ± 0.14	4.56^e^ ± 0.09
100% ethanol	2.24^a^ ± 0.19	2.79^a^ ± 0.04	2.14^a^ ± 0.09	2.29^a^ ± 0.07	1.63^a^ ± 0.04	1.86^a^ ± 0.09	0.85^a^ ± 0.03
80% ethanol	4.66^c^ ± 0.04	4.20^b^ ± 0.16	3.47^b^ ± 0.22	4.13^b^ ± 0.02	3.35^c^ ± 0.09	3.94^d^ ± 0.19	3.32^d^ ± 0.10
Water	2.61^a^ ± 0.06	2.58^a^ ± 0.07	2.23^a^ ± 0.05	2.47^a^ ± 0.11	2.54^b^ ± 0.08	2.31^b^ ± 0.09	1.95^b^ ± 0.08
80% acetone	8.43^e^ ± 0.31	7.49^d^ ± 0.31	5.51^d^ ± 0.10	6.71^d^ ± 0.12	5.92^f^ ± 0.25	6.85^f^ ± 0.21	6.85^f^ ± 0.14

a,b,c,d,e,f Values followed by the same letter within one variety (in columns) are not statistically different at *P* < 0.05, ANOVA, Tukey HSD.

The second most effective solvent for polyphenol extraction was acid methanol, for which the values were more balanced among varieties and ranged from 4.56 (Zoe) to 5.71 mg GAE/g (Darja). In the tests with 80% methanol and 80% ethanol, results are comparable for six varieties out of seven; for the Zamira variety, 80% methanol was more effective than 80% ethanol. The TPC in 80% methanol ranged from 3.20 (Zoe) to 4.80 mg GAE/g (Darja), and the values in 80% ethanol ranged from 3.32 (Zoe) to 4.66 mg GAE/g (Darja). The 100% methanol solvent performed better than water for all varieties but worse than 80% methanol for the Darja, Zamira, Zita, and Zoe varieties. The TPC in 100% methanol ranged from 2.48 (Zoe) to 4.11 mg GAE/g (Darja). The TPC values in water ranged from 1.95 (Zoe) to 2.61 mg GAE/g (Darja). For the Darja, Harpe, Kora, and Panda varieties, the TPC content from water extraction was comparable with that from extraction in 100% ethanol. For the Zamira, Zita, and Zoe varieties, TPC was significantly higher when extracted with water than with 100% ethanol.

Concerning the efficacy of acetone as a solvent for TPC extractions, our results are consistent with a study published by Sun et al. (18), in which TPC in buckwheat using five different solvents was compared. The authors arranged the solvents in order from highest to lowest efficacy: acetone > methanol = ethanol > butanol = ethyl acetate. The only slight difference between the results is that our results showed better efficacy of methanol compared with ethanol. This was especially true for a 100% concentration of these solvents as TPC was significantly higher using methanol for all studied varieties. Acetone was also a superior solvent for polyphenol extraction from wheat samples in a study published by Zhou et al. (13). Inglett et al. investigated the TPC of commercial buckwheat flours using three solvents (50 and 100% ethanol and water) (19). In this study, the efficacy of solvents was arranged in the following order: 50% ethanol > water > 100% ethanol. In our study, 80% ethanol gave better results for all buckwheat varieties compared with water. However, a comparison between water and 100% ethanol suggests the influence of the buckwheat variety because water was a better solvent for three varieties, but there was no difference between these two solvents for four varieties. Zielinski et al. compared the TPC of extracts from cereals, including buckwheat, using 80% methanol and water as solvents (20). For all studied cereals, extracts using 80% methanol yielded a higher value of TPC than extracts in water did, which is fully consistent with the results of our study.

### Effect of temperature

Two approaches demonstrated the influence of temperature on TPC extraction. The results are summarized in Table 3. Table 3A shows the comparison of extraction by 80% methanol, in which the first sample was incubated overnight at room temperature and the second sample was incubated for 2 h at 80°C. Results revealed that the second approach was more effective for all tested varieties. The TPC obtained by extraction at 80°C ranged from 4.78 (Zoe) to 8.37 mg GAE/g (Darja). The values are 29% (Zamira) and 74% (Darja) higher than the corresponding values obtained at room temperature. Higher molecular movement speeds at higher temperatures may allow phenolic compounds to diffuse more quickly from sample cells (21). Thus, procedures utilizing a higher temperature and 80% methanol have given only slightly lower TPCs than extraction by 80% acetone, which was the most effective room-temperature solvent. This is consistent with a study published by Inglett et al. (8), which described the influence of increased temperature on TPC in buckwheat extraction by three solvents. Between 23°C and 50°C, the values of phenol content increased but only slightly, and the values doubled at 100°C. The same conclusions were published by Hodzic et al. (22), who studied water extracts of buckwheat. Xu et al. determined 80°C as an optimal temperature for phenolic extraction because TPC values decreased between 80°C and 100°C due to the decomposition and oxidation of some polyphenols at higher temperatures (21).

**Table 3:** The total polyphenol content of seven varieties of common buckwheat obtained at different temperatures

		TPC (mg GAE/g)						
		Darja	Harpe	Kora	Panda	Zamira	Zita	Zoe
**A**	80% metanol	4.80^a^ ± 0.04	4.12^a^ ± 0.12	3.44^a^ ± 0.10	4.13^a^ ± 0.23	3.79^a^ ± 0.09	4.11^a^ ± 0.26	3.20^a^ ± 0.23
	80% methanol (80°C)	8.37^b^ ± 0.81	6.14^b^ ± 0.16	5.09^b^ ± 0.28	5.42^b^ ± 0.19	4.89^b^ ± 0.14	5.37^b^ ± 0.13	4.78^b^ ± 0.21
**B**	0.1% HCl in methanol	5.71^a^ ± 0.12	5.50^b^ ± 0.13	4.59^b^ ± 0.15	5.41^b^ ± 0.20	4.95^a^ ± 0.21	5.38^a^ ± 0.14	4.56^a^ ± 0.09
	0.1% HCl in methanol (−20°C)	5.86^a^ ± 0.08	4.65^a^ ± 0.23	4.12^a^ ± 0.10	4.76^a^ ± 0.09	4.94^a^ ± 0.08	5.00^a^ ± 0.10	4.36^a^ ± 0.27

(A) Extraction by 80% methanol, in which the first sample was incubated overnight at room temperature and the second sample was incubated at 80°C for 2 h. (B) Extraction by 0.1% HCl in methanol, in which the first sample was incubated overnight at room temperature and the second sample was incubated overnight at −20°C. Results are the means of four replications ± SD.

a,b Values followed by the same letter within one variety and the same temperature are not statistically different at *P* < 0.05, ANOVA, Tukey HSD.

Table 3B compares extraction by acid methanol (0.1% HCl in methanol), in which samples were incubated overnight at room temperature or −20°C. Lower temperatures do not influence the TPC as much as higher temperatures do. The TPC values obtained at −20°C were significantly lower for three varieties (Harpe, Kora, and Panda), and no influence of lower temperatures was observed for the remaining four varieties.

### Effect of hydrolysis

As mentioned previously, aqueous-organic extraction releases only ‘extractable’ polyphenols. The solid residues after extraction are not generally considered a source of bioactive compounds. Nevertheless, a significant number of phenolic compounds remain in residues after extractions and are marked as ‘nonextractable’ or ‘bound’ polyphenols. Proanthocyanins, phenolic acids, and hydrolyzable tannins are closely associated with the cell wall and are constituents of dietary fibre (23). Bound polyphenols are concentrated mainly in the outer layer of seeds, that is, in the brans (24). These compounds are released by alkaline, acid, or enzymatic hydrolysis. The hydrolysis method affects the phenol content and the phenolic compound profile because complex structures may decompose during hydrolysis. A general hypothesis in the literature is that TPC values will be higher as a result of hydrolysis (25–27).

In our research, two different modifications (classical and sequential) of both alkaline and acid hydrolysis were tested. In classical hydrolysis, the sample was directly heated in an alkaline or acidic solution, whereas in sequential hydrolysis, the sample was first extracted overnight by 80% methanol and then heated in an alkaline or acidic solution. The results are summarized in Table 4. Alkaline hydrolysis was more effective than acid hydrolysis. In both classical and sequential modifications, acid hydrolysis gave an average of one-third lower TPC values than alkaline hydrolysis. A comparison of results from Tables 2 and 4 clarifies that higher TPC values are obtained in hydrolysis than in solvent extraction. TPC values for acid and alkaline hydrolysis were approximately 1.9 and 2.8 times higher than those for 80% acetone (the most effective extraction solvent), respectively. Both classical and sequential hydrolysis methods can provide similar results, which was demonstrated in most buckwheat varieties, except variety Darja, as shown in Table 4. This study suggests that previously published results from literature may underestimate the TPC due to the exclusion of bound polyphenols in cell walls that are released only through hydrolysis.

**Table 4:** The total polyphenol content of seven varieties of common buckwheat obtained by acid and alkaline hydrolysis, both methods using classical and sequential (S) modification (mg GAE/g)

	TPC (mg GAE/g)						
	Darja	Harpe	Kora	Panda	Zamira	Zita	Zoe
Acid hydrolysis	17.01^a^ ± 0.97	12.43^a^ ± 0.12	11.60^a^ ± 0.32	11.33^ab^ ± 1.04	11.30^a^ ± 0.38	11.16^a^ ± 0.51	11.95^a^ ± 0.94
Acid hydrolysis (S)	19.97^b^ ± 1.03	14.13^a^ ± 0.74	10.72^a^ ± 0.30	10.92^a^ ± 0.08	10.52^a^ ± 0.37	12.52^a^ ± 0.20	12.35^a^ ± 0.67
Alkaline hydrolysis	32.96^c^ ± 2.15	21.05^b^ ± 1.24	16.93^b^ ± 0.93	15.40^c^ ± 1.07	17.07^b^ ± 0.42	16.80^b^ ± 0.80	18.28^b^ ± 0.67
Alkaline hydrolysis (S)	31.26^c^ ± 1.02	19.79^b^ ± 1.22	16.32^b^ ± 1.13	14.07^bc^ ± 0.77	17.17^b^ ± 0.25	15.77^b^ ± 0.76	19.37^b^ ± 1.14

Results are the means of four replications ± SD.

a,b,c Values followed by the same letter within one variety and the same temperature are not statistically different at *P* < 0.05, ANOVA, Tukey HSD.

### TPC in different common buckwheat varieties

Significant differences in physical properties and nutritional compositions were detected among buckwheat varieties. A large genetic variation was observed, for example, in thousand kernel weight and the contents of protein, fibre, and free and total polyphenols (28–30). The question remains as to the expected response for individual buckwheat varieties to different extraction solvents and procedures for TPC extraction.

**Fig. 1:**
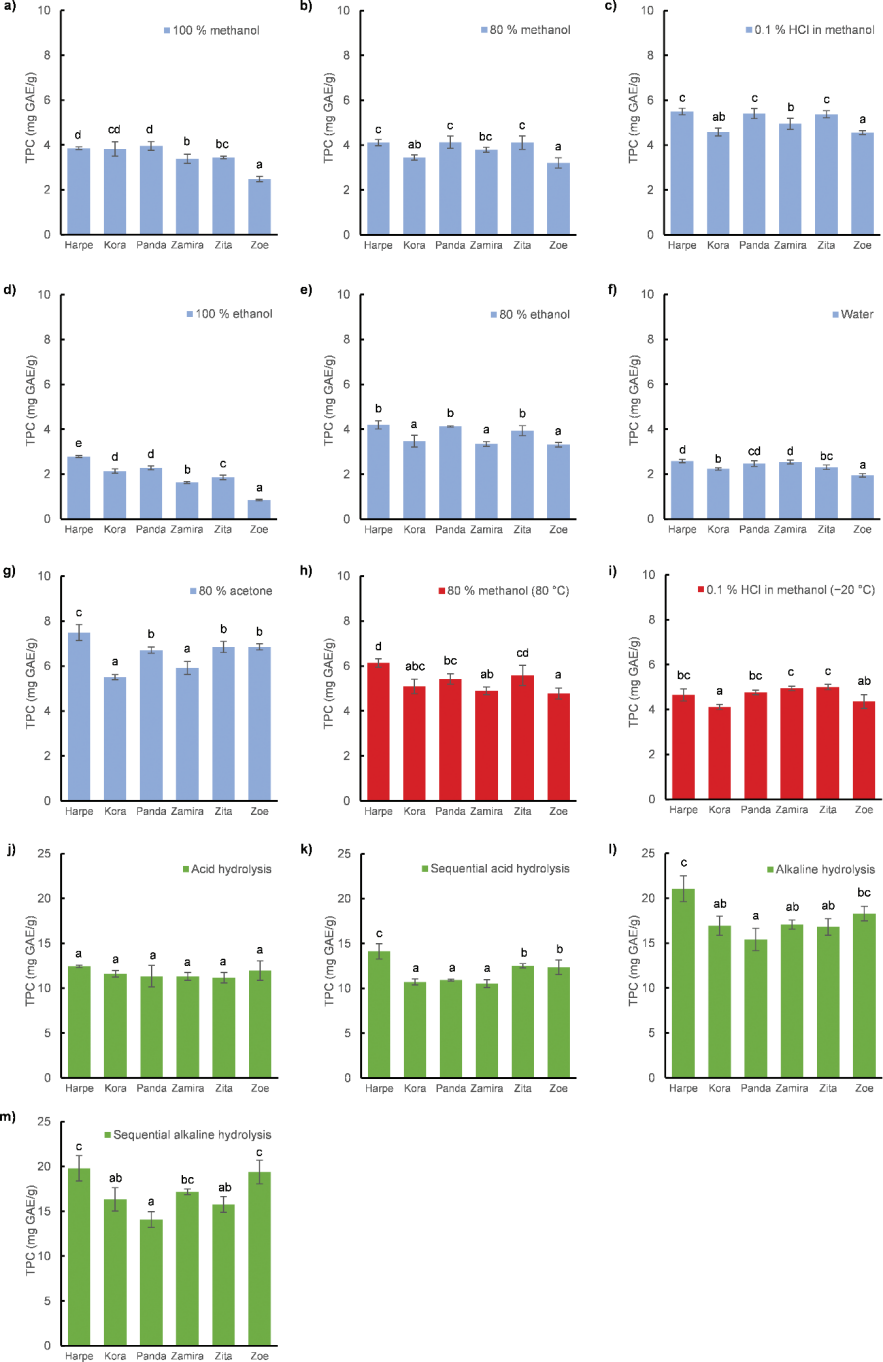
The total phenol content (TPC) of six common buckwheat varieties in different aqueous-organic solvents (a–g), the influence of temperature (h–i), and the influence of hydrolysis (j–m).

TPC has been proven to potentially be largely influenced by environmental conditions, including growing location (31–33). Therefore, six common buckwheat varieties (Harpe, Kora, Panda, Zamira, Zita, and Zoe) grown at the same locality under the same growing conditions were only used for comparative analysis. The Darja variety was excluded from statistical evaluation because of its different origin. Our results suggest that TPC is influenced by both the variety and extraction method (Fig. 1). In aqueous-organic solvent extraction (Fig. 1a–g), the Harpe variety had the highest TPC in all solvents either as the only variety (using 100% ethanol and 80% acetone) or together with some other varieties. That is, using 80% ethanol, 80% methanol, and acid methanol, the highest TPC was found for the Harpe, Panda, and Zita varieties. Using 100% methanol, the highest TPC was found for the Harpe and Panda varieties and using water, the highest TPC was found for the Harpe and Zamira varieties. For most of the solvents, the Zoe variety exhibited the lowest TPC, either exclusively (in 100% methanol, 100% ethanol, and water), together with the Kora and Zita varieties (in 80% ethanol), or the Kora variety (in 80% methanol and 0.1% HCl in methanol). The only solvent providing different results was 80% acetone, for which the lowest TPC was found for the Kora and Zamira varieties, whereas the Zoe variety exhibited medium TPC content together with the Zita and Panda varieties. The increase in temperature led to an increase in TPC content, but the order of TPC values among the varieties did not change significantly (Fig. 1h–i). Using classical acid hydrolysis, there were no differences in the TPC content between varieties (Fig. 1j). However, the results of sequential acid hydrolysis (Fig. 1k) categorized the varieties into three different groups with the highest, middle, and lowest TPC values as follows: the Harpe variety, followed by the Zita and Zoe varieties, and lastly, the Kora, Panda, and Zamira varieties, respectively. The ranking of the varieties according to their TPC obtained by classical and sequential alkaline hydrolysis was similar with slight differences (Fig. 1l–m). The highest TPC was found for the Harpe and Zoe varieties, and the lowest was found for the Panda variety.

The obtained results showed that the choice of method matters when testing varieties for TPC, for example, for breeding purposes, because different information may be available in different methods. Some of the methods in our study did not detect any TPC differences between varieties, and other methods classified varieties according to TPC into up to four groups. Using 100% methanol and water as solvents, the highest TPC values were found for the Harpe and Panda varieties. Therefore, these varieties are assumed to be a good source of free or soluble conjugated polyphenols. When alkaline hydrolysis was used, the Harpe variety consistently demonstrated the highest TPC, whereas the TPC differed for the other varieties. The biggest shift in the TPC values occurred in the case of variety Zoe for which the TPC were the lowest for almost all solvents and increased when hydrolysis was applied. In the case of sequential alkaline hydrolysis, the Zoe TPC was comparable to that of the variety Harpe. This could be explained by the higher content of bound polyphenols which are released by hydrolysis.

## Conclusion

The results showed that the TPC is significantly dependent on the extraction solvent and the proven effectiveness of the solvents can be arranged in the following order: 80% acetone > 0.1% HCl in methanol > 80% methanol = 80% ethanol > 100% methanol > water = 100% ethanol. The temperature was another affecting factor, the TPC after extraction in 80% methanol at 80°C was significantly higher than after extraction at room temperature. All hydrolysis procedures proved to be more effective for phenolic extraction than solvent extraction. The highest TPC was obtained by alkaline hydrolysis for all varieties, without significant differences between classical and sequential modifications. Except for classical acid hydrolysis, all other solvents and extraction approaches identified significant differences in the TPC between buckwheat varieties. The results showed that the choice of the method matters when testing buckwheat varieties for TPC, for example, for breeding and food use purposes, because different information may be obtained by different methods. Some of the methods used in our study did not detect TPC differences between varieties, whereas other methods classified varieties according to TPC into up to four groups. This is probably the specific composition of extractable and bounded polyphenols of each variety, which affects its TPC response to different solvents and procedures. However, to support this affirmation, further research focusing on the detailed composition of the polyphenol spectrum of the individual buckwheat varieties would be needed.

## Data Availability

The data sets generated during the current study are available upon request, please contact the contributing authors.
